# Early first trimester diagnosis and management of rudimentary horn pregnancy: A rare case report

**DOI:** 10.1016/j.radcr.2025.05.074

**Published:** 2025-06-27

**Authors:** Youssef Essebbagh, Khadija Errmili, Fatima Z. Belouazza, Aziz Slaoui, Najia Zeraidi, Aziz Baidada

**Affiliations:** Gynaecology-Obstetrics and Endoscopy Department, Maternity Souissi, University Hospital Center IBN SINA, University Mohammed V, Rabat, Morocco

**Keywords:** Rudimentary horn pregnancy, Ectopic pregnancy, Unicornuate uterus, First trimester, Ultrasonography

## Abstract

Rudimentary horn pregnancy (RHP) is a rare and potentially life-threatening form of ectopic pregnancy that occurs in the context of a unicornuate uterus resulting from incomplete development of 1 Müllerian duct. We present the case of a 31-year-old nulligravida woman diagnosed at 8 weeks and 2 days of amenorrhea with an RHP, initially suspected via pelvic ultrasound and confirmed by laparoscopy. The patient underwent surgical resection of the rudimentary horn containing the pregnancy, with an uneventful postoperative course. RHP often presents with nonspecific symptoms, making early diagnosis difficult, and carries a high risk of uterine rupture and placenta accreta if left untreated. This case highlights the critical role of early ultrasonographic evaluation and emphasizes the importance of prompt surgical intervention to prevent severe maternal morbidity.

## Introduction

A unicornuate uterus with a rudimentary horn is a rare congenital anomaly caused by complete or partial failure of one of the Müllerian ducts to develop. Rudimentary horn pregnancy is an exceptionally rare condition that poses significant diagnostic challenges. Its subtle and nonspecific clinical presentation, coupled with decreasing sensitivity of ultrasonography as pregnancy progresses, complicates early detection [1].

In this report, we present the case of a 31-year-old who arrived at the emergency room with complaints of pelvic pain and amenorrhea lasting 8 weeks and 2 days. Ultrasonography raised suspicion of a rudimentary horn pregnancy, which was subsequently confirmed through diagnostic laparoscopy. Through this case and a review of the literature, we emphasize the key features of this condition, particularly the diagnostic methods, therapeutic management, and prognosis of this rare complication.

## Case presentation

A 31-year-old woman, gravida 2, para 0, with no notable medical or surgical history, presented at 8 weeks and 2 days of amenorrhea following a positive urine pregnancy test. Her obstetric history included an early miscarriage 1 year prior, managed without surgical intervention. She denied any chronic illnesses, medication use, or known congenital anomalies. There was no family history of uterine malformations, infertility, or recurrent pregnancy loss.

The patient presented to the emergency department with recent onset of lower abdominal pain, described as mild and intermittent. She reported no vaginal bleeding, nausea, or systemic symptoms. She had not undergone any prior ultrasound during the current pregnancy.

On examination, she was alert and hemodynamically stable, with normal blood pressure and heart rate. Abdominal examination was unremarkable, and pelvic examination revealed a closed cervix without vaginal discharge or bleeding. Bimanual examination showed an enlarged, nontender uterus with localized left-sided pelvic tenderness.

**Fig. 1:**
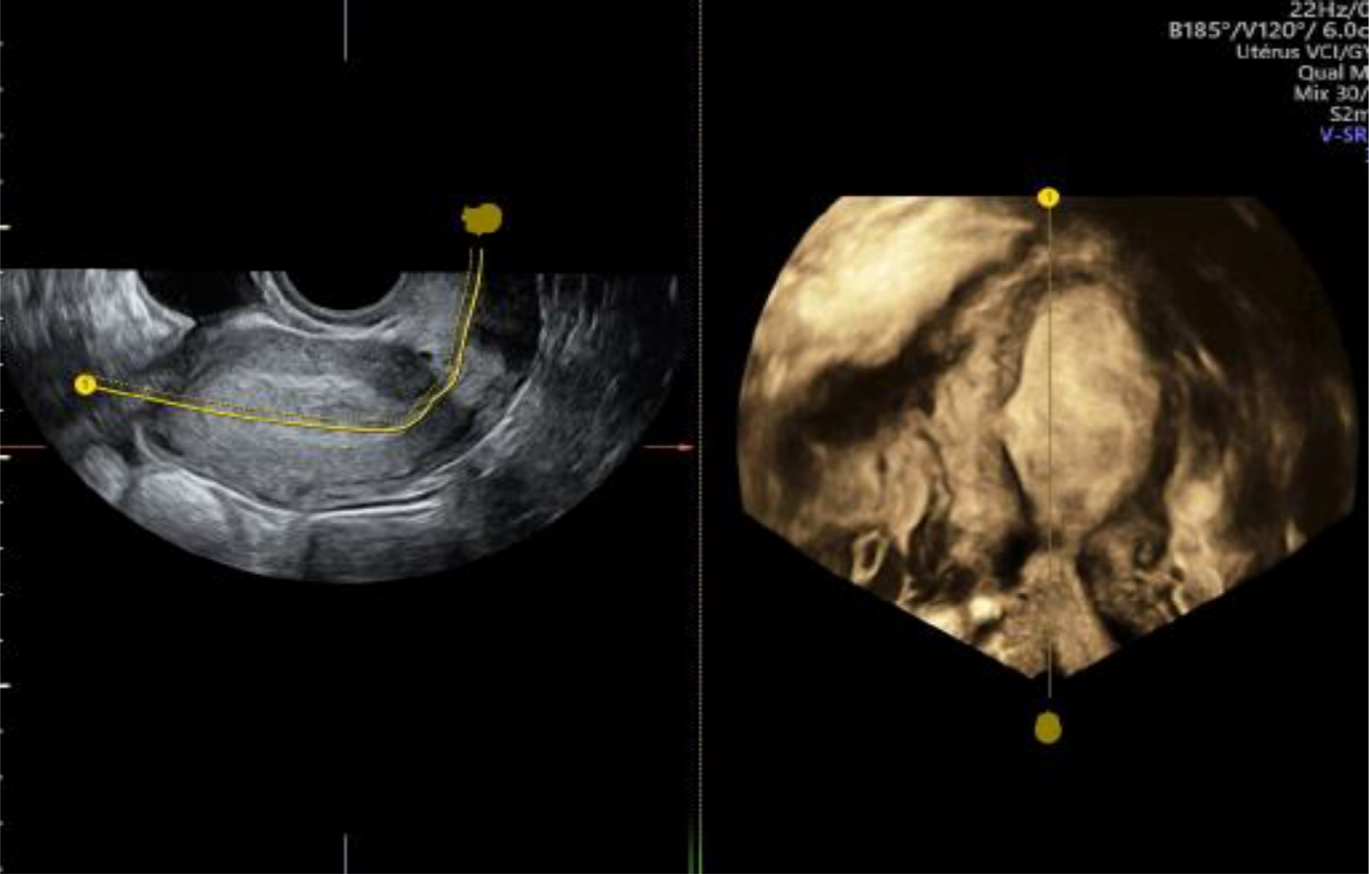
Enovaginal ultrasound 3D empty unicorn uterus.

**Fig. 2:**
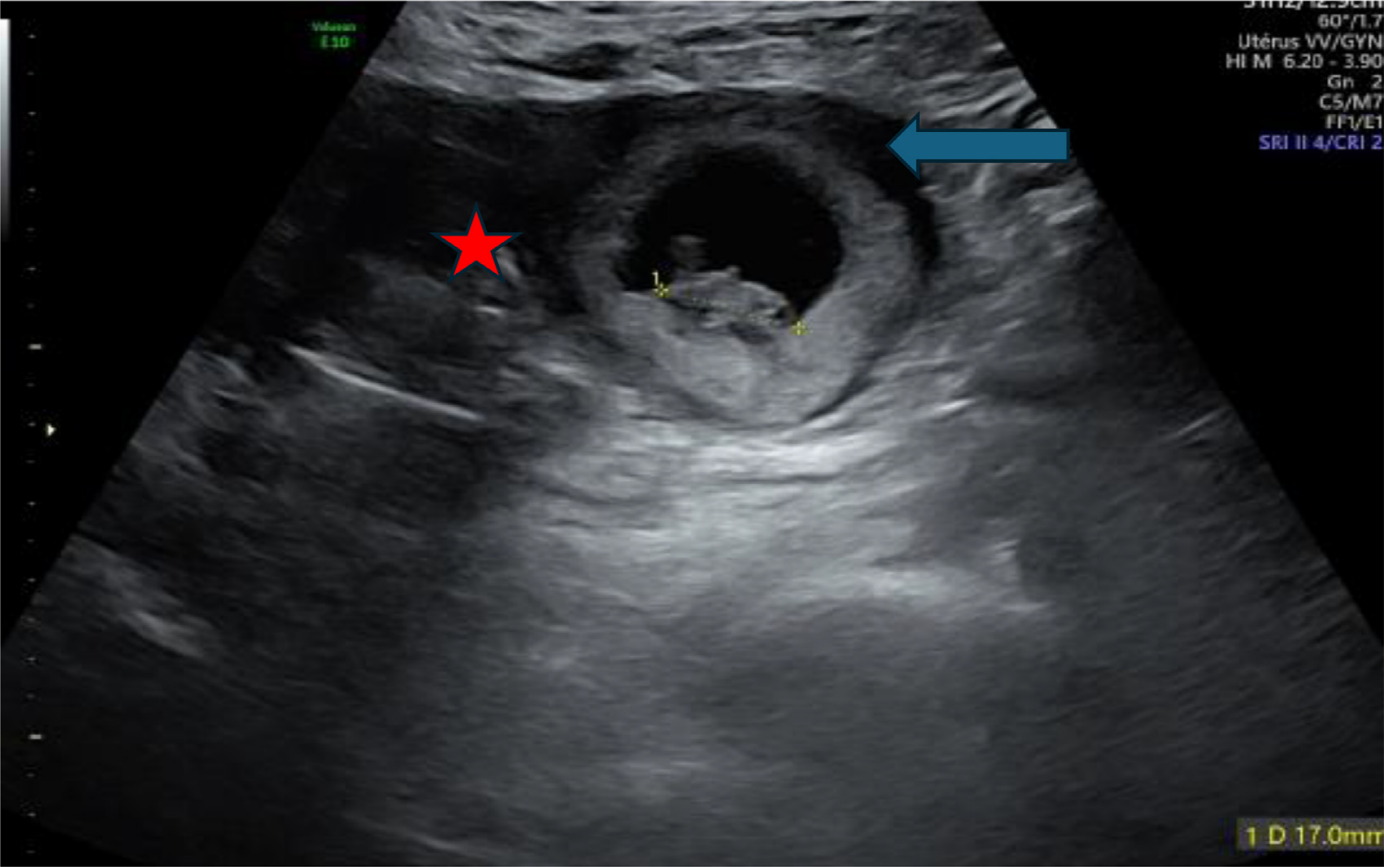
Empty uterus: red star, pregnancy: blue arrow.

Pelvic transvaginal ultrasound revealed a likely unicornuate uterus with a noncommunicating rudimentary horn on the left side. A gestational sac with a live embryo was visualized within the rudimentary horn, surrounded by a ring of myometrium and separated from the main uterine body by a fibrous band. Cardiac activity was present, and the crown-rump length measured 17 mm, corresponding to 8 weeks and 2 days of gestation. The endometrial cavity of the main uterus was empty. Both ovaries appeared normal, with no adnexal masses or free fluid noted. Renal ultrasound confirmed both kidneys in their normal positions with no anomalies (Fig. 1, Fig. 2).

Laboratory evaluation included a serum β-hCG level of 10,000 IU/mL, consistent with the gestational age Complete blood count was within normal limits, with hemoglobin at 13.2 g/dL, white blood cell count at 7500/mm³, and platelet count at 250,000/mm³. Renal function tests were normal, with serum creatinine at 0.8 mg/dL and blood urea at 12 mg/dL.

**Fig. 3:**
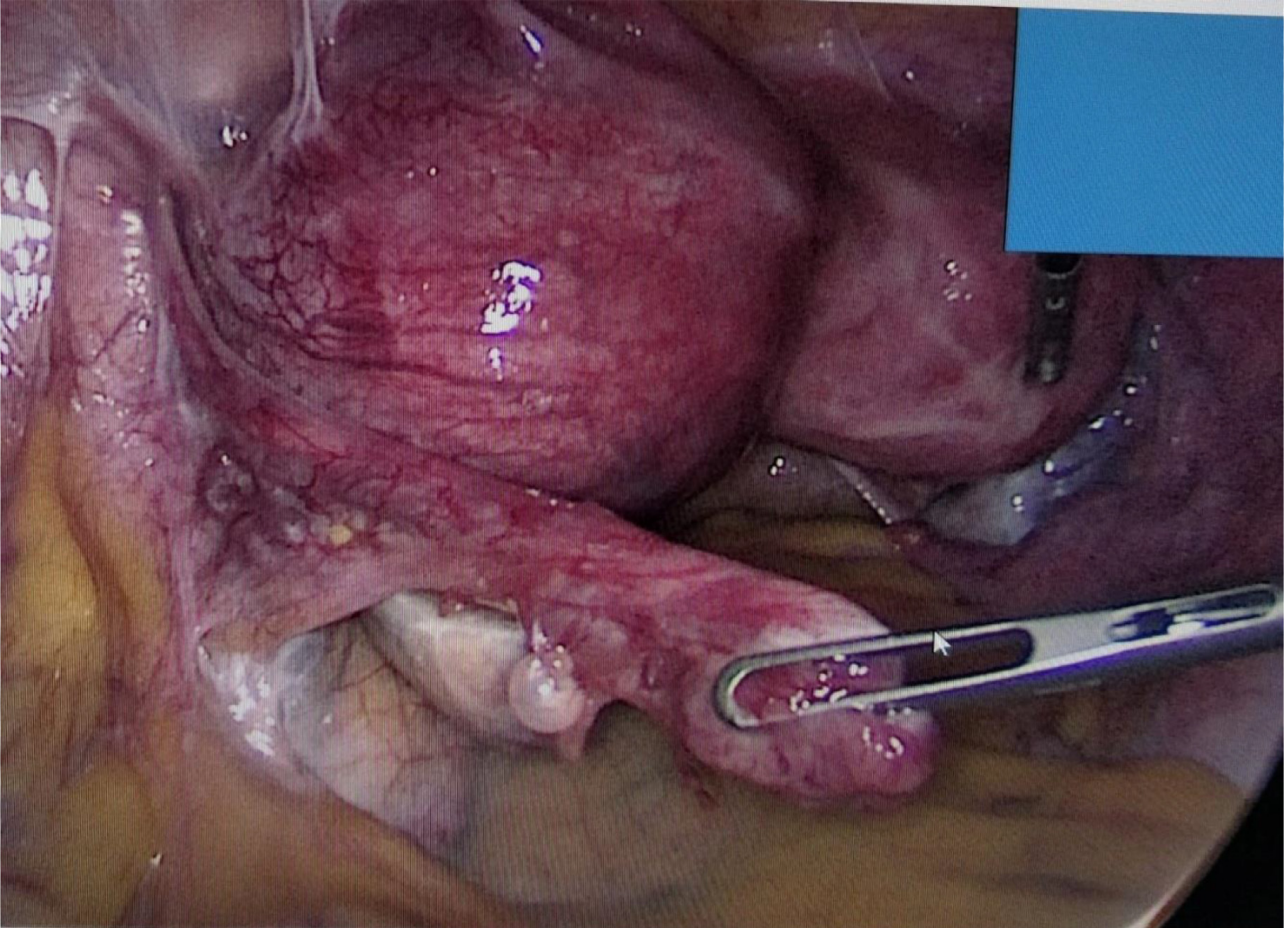
Intraoperative view of a pregnancy in the rudimentary horn of a unicornuate uterus.

Given the high suspicion of a rudimentary horn pregnancy (RHP) and the associated risk of rupture, the patient was scheduled for laparoscopic exploration. Intraoperatively, a noncommunicating left rudimentary horn was confirmed with a distended gestational sac. The horn was attached to the unicornuate uterus via a fibrous band. A complete laparoscopic resection of the rudimentary horn and ipsilateral fallopian tube was performed without intraoperative complications (Fig. 3).

The patient had an uneventful postoperative course and was discharged on the second postoperative day. Histopathological examination confirmed the diagnosis of pregnancy within the excised rudimentary horn.

She was followed regularly at our institution for 2 years and later conceived spontaneously. Her subsequent pregnancy progressed without complications, and a planned cesarean section was performed at 38 weeks of gestation. A healthy infant was delivered, and intraoperative findings revealed no evidence of uterine dehiscence or structural abnormalities.

## Discussion

In our case, the diagnosis of a rudimentary horn pregnancy (RHP) highlights the clinical significance of this rare Müllerian anomaly. A unicornuate uterus results from the incomplete development of 1 Müllerian duct during embryogenesis. This anomaly is frequently associated with varying degrees of rudimentary horn formation, which arises when one of the paramesonephric ducts develops only partially or fails to canalize. The resulting rudimentary horn may or may not communicate with the main uterine cavity. Classified as a Class II Müllerian anomaly by the American Society for Reproductive Medicine (ASRM), this condition predisposes patients to several gynecological and obstetric complications. In particular, RHP is a rare and potentially life-threatening form of ectopic pregnancy. Due to the limited distensibility and poor musculature of the rudimentary horn, rupture is common—typically occurring in the second trimester—and can lead to severe intra-abdominal hemorrhage. The estimated prevalence of RHP ranges from 1 in 76,000 to 1 in 150,000 pregnancies, with a maternal mortality rate of up to 0.5%, most often due to delayed or missed diagnosis. This underscores the importance of early imaging and high clinical suspicion in patients presenting with atypical pregnancy symptoms and known uterine anomalies [[1], [2], [3]].

A lack of experience with diagnostic criteria and vague symptomatology are the primary factors contributing to the increased risk of complications during rudimentary horn pregnancies. The first trimester is particularly crucial for timely preoperative diagnosis, as demonstrated in our case. After this period, diagnosing a rudimentary horn pregnancy becomes significantly more challenging [4,5].

Early diagnosis of RHP remains challenging. While ultrasonography (US) can be helpful in diagnosing RHP, its sensitivity is only 26% and tends to decrease as the pregnancy progresses.

Tsafrir et al. have suggested the following criteria for the ultrasonographic diagnosis of rudimentary horn pregnancy [6,7]:

1. A pseudo-pattern of an asymmetrical bicornuate uterus,
2. Absent visual continuity of tissue surrounding the gestational sac and the uterine cervix,
3. The presence of myometrial tissue surrounding the gestational sac.

Although the diagnosis of rudimentary horn pregnancy (RHP) was made using ultrasonography, performing a preoperative MRI could have been beneficial. MRI offers superior soft tissue contrast and multiplanar imaging, which may have provided more conclusive evidence of the uterine anomaly and improved presurgical planning accuracy.

Despite the risk of rupture, which is the natural course of RHP, pathological placentation is also a common complication that has been previously described. Placenta accreta is commonly observed in pregnancies involving a rudimentary uterine horn. Based on previously reported cases in the literature, Oral and al. estimated the prevalence of placenta accreta in rudimentary uterine horn pregnancies to be 11.9% [[8], [9], [10], [11]].

Given the hemorrhagic risk associated with placenta accreta and the potential for spontaneous uterine rupture due to the thin myometrium, immediate surgical intervention to remove the pregnant rudimentary uterine horn should be carried out as soon as the diagnosis is confirmed [7,12].

The standard treatment for rudimentary horn pregnancy is the surgical excision of both the pregnancy and the rudimentary horn. This procedure serves both as a therapeutic and diagnostic approach for RHP. When the pregnancy occurs in the rudimentary horn during the first trimester, laparoscopic excision is recommended, as demonstrated in our case [7].

To optimize future obstetrical outcomes, preconception counseling is essential. Patients should be advised about these risks and the importance of planning pregnancies in conjunction with a healthcare provider. Close antenatal monitoring, including regular ultrasounds and potential hospitalization in the third trimester, is recommended to detect and manage complications promptly. Delivery in a well-equipped facility with surgical capabilities is crucial, as cesarean delivery is often preferred to mitigate the risk of uterine rupture during labor [[8], [9], [10]], Our patient was followed up during a subsequent spontaneous pregnancy carried to term without complications. An elective cesarean section was performed at 38 weeks of gestation, with no evidence of uterine dehiscence.

Most reported cases of rudimentary horn pregnancy (RHP) are diagnosed during the second trimester, often following horn rupture due to delayed symptom onset or initial misdiagnosis. In contrast, the present case was identified in the first trimester through early imaging, which enabled timely surgical intervention and likely prevented catastrophic hemorrhagic complications. This highlights the critical role of early radiological evaluation in patients with known or suspected Müllerian anomalies. Furthermore, the patient’s subsequent spontaneous intrauterine pregnancy and successful delivery are particularly noteworthy. Fertility preservation following excision of a rudimentary horn is not always guaranteed, as many patients experience infertility or recurrent pregnancy loss. Compared to previously published cases, this favorable reproductive outcome adds valuable clinical insight and reinforces the importance of individualized surgical management and close follow-up [[8], [9], [10]].

Fertility prospects in patients with a unicornuate uterus remain generally favorable, as one functioning ovary and fallopian tube are typically present. However, the removal of the rudimentary horn and its associated fallopian tube slightly reduces reproductive potential. Advanced fertility interventions, such as assisted reproductive technologies (ART), may be considered in cases of infertility [9,12].

This case underscores the importance of training healthcare providers to recognize and manage RHP, particularly in resource-limited settings where access to advanced imaging and specialized care may be restricted. Integrating 3-dimensional ultrasound into early pregnancy units and providing targeted education for clinicians could significantly enhance diagnostic accuracy and patient outcomes.

## Conclusion

Rudimentary horn pregnancy (RHP) is a rare and challenging condition that requires a high level of clinical suspicion for timely diagnosis. Despite its rarity, early detection is crucial to prevent complications such as rupture, hemorrhage, and placenta accreta. In this case, the diagnosis of RHP was made through ultrasonography. Although the sensitivity of ultrasound for diagnosis is low, it can play an important role in diagnosing RHP, especially in the early weeks of pregnancy. Therefore, obstetricians should consider this condition, particularly in patients with a history of pelvic chronic pain, endometriosis, or infertility. The absence of visual continuity between the tissue surrounding the gestational sac and the uterine cervix may be the most significant criterion to alert the obstetrician. Surgical excision of the pregnancy and rudimentary horn remains the gold standard for treatment.

## Ethical approval

Ethical approval is not applicable. The case report is not containing any personal information.

## Patient consent

Written informed consent was obtained from the patient for the publication of this case report and any accompanying images. The patient has reviewed the manuscript and agrees to the inclusion of their clinical details and relevant diagnostic findings. Efforts have been made to maintain patient anonymity, and no identifying information is included in this report.

## Author contributions

**Youssef Essebbagh** contributed to patient care, conception of the case report, acquiring and interpreting the data, undertaking the literature review and drafting the manuscript.

**Khadija Errmili** contributed to the conception of the case report, acquiring and interpreting the data and and revising the article critically for important intellectual content.

**Fatemzahra Belouza** contributed to patient care, drafting the manuscript, undertaking the literature review. **Aziz slaoui** contributed to patient care, drafting the manuscript and acquiring and interpreting the data. **Najia Zeraidi** contributed to undertaking the literature review and revising the article critically for important intellectual content. **Aziz Baidada** contributed to undertaking the literature review and revising the article critically for important intellectual content. All authors approved the final submitted manuscript.
